# A positive association between hunger in childhood and frailty in old age: Findings from the Chinese longitudinal healthy longevity survey

**DOI:** 10.3389/fmed.2022.955834

**Published:** 2022-10-24

**Authors:** Tianjing Gao, Siyue Han, Guangju Mo, Qing Sun, Min Zhang, Huaqing Liu

**Affiliations:** ^1^School of Public Health, Bengbu Medical College, Bengbu, China; ^2^School of Health Management, Bengbu Medical College, Bengbu, China

**Keywords:** childhood, China, frailty, hunger, older people

## Abstract

**Background:**

Childhood hunger not only directly affects the physical and mental health of children and adolescents but also has a long-term negative effect on later health outcomes. In this cross-sectional study, we used a nationally representative Chinese sample to examine the relationship between hunger in childhood and frailty in older adults.

**Materials and methods:**

The data were obtained from the 2018 Chinese Longitudinal Healthy Longevity Survey. The frailty index with 44 health deficits was used to identify frailty. Childhood hunger was measured by the question “Did you often go to bed hungry as a child?” Insurance status was categorized as New Rural Cooperative Medical Scheme (NRCMS), Urban Basic Medical Insurance Scheme (UBMIS), others, and no insurance. Multivariate logistic regression analysis was performed to estimate the adjusted relationship between childhood hunger and frailty.

**Results:**

A total of 7,342 older people aged 65 years and older were analyzed in this study. Older people who experienced childhood hunger were more likely to have frailty than those who did not (OR = 1.13, 95% CI: 1.02–1.26), after adjustment for sociodemographic characteristics, family/social support, socioeconomic status, insurance status, and health behaviors. The association of childhood hunger with frailty was found in the 65–79 years group (OR = 1.21, 95% CI: 1.03–1.43), women (OR = 1.25, 95% CI: 1.08–1.45), individuals with rural residence (OR = 1.16, 95% CI: 1.03–1.31), agricultural work (OR = 1.16, 95% CI: 1.00–1.34), financial dependence (OR = 1.18, 95% CI: 1.02–1.37), and those participating in NRCMS (OR = 1.35, 95% CI: 1.16–1.56). Participants with hunger in childhood who were 80 years or older (OR = 0.80, 95% CI: 0.65–0.98) had lower odds of frailty. NRCMS (OR = 1.42, 95% CI: 1.02–1.98) showed increased odds of childhood hunger-related frailty.

**Conclusion:**

Exposure to hunger during childhood is linked to frailty among older adults, and age, financial support, and insurance status may mediate this relationship. Targeted interventions and policies to address frailty in older adults should be implemented.

## Introduction

Frailty is a modern geriatric syndrome among older adults and is one of the most serious global public health challenges we will face in the next century (1). It reflects a multifactorial syndrome that includes physical, psychological, and social deficits that accumulate during the aging process, loss of reserves, and decreased resistance to stress and is linked with a high risk of adverse health-related outcomes, such as decreased functional capacity, falls, delirium, hospitalization, and death (2). Research (3) shows that frailty is reversible, and health promotion, nutrition, and physical and social support interventions can be used to treat and delay frailty.

China experienced the Great Leap Forward Famine in 1959–1961. Most Chinese people aged over 65 years today have experienced famine in their early life (4). Early life food deprivation has been found to be an important risk factor of negative health outcomes (5) and increase the risk of developing obesity, diabetes, hypertension, and other diseases in adulthood (6). Childhood experiences of hunger are common among older people, and understanding the impact of hunger on individuals can be particularly enlightening (7). There is growing evidence that traumatic events in childhood may have an impact on health throughout the life course (7).

The link between childhood conditions and health in later life might be explained by the theory of cumulative disadvantage/advantage, which places individual trajectories under the context of structural factors that might ameliorate or exacerbate previous disadvantages/advantages, and further influences individual health in later life and population-level inequality (8). The three aspects of the cumulative disadvantage/advantage hypothesis can be tested in the context of frailty as follows. First, to examine the relationship between childhood hunger and frailty by age, sex, and residence. Second, to examine the role of adulthood socioeconomic conditions (e.g., education, occupation, and financial support) in the association between childhood hunger and frailty at older age. Third, to evaluate the role of insurance status in the association between child hunger and frailty. Relating factors in the life course to frailty will increase our understanding of the social origins of frailty (9). Therefore, this study aimed to evaluate the association between childhood hunger and frailty in older people and to determine the roles of sociodemographic characteristics and socioeconomic status in this association.

## Materials and methods

### Study sample

Data for the present study were obtained from the seventh wave of the Chinese Longitudinal Healthy Longevity Survey (CLHLS) in 2018, which was conducted by Peking University and the Chinese Center for Disease Control and Prevention. The CLHLS is a nationally representative survey that aims to understand the health status of older adults and related biological, behavioral, and social factors in China. A multi-stage disproportionate and targeted random sampling was adopted. Approximately 50% of counties/districts were randomly selected from 23 out of the 31 provinces of mainland China, in which all centenarians who volunteered to participate were interviewed. For each centenarian interviewee, one non-agenarian, one octogenarian, and three participants aged 65–79 years were matched nearby in the same street, village, or town. All information was obtained in participants’ homes through face-to-face interviews using internationally compatible questionnaires by trained investigators. The CLHLS study was approved by the Research Ethics Committee of Peking University (IRB00001052-13074), and all participants provided written informed consent.

**FIGURE 1:**
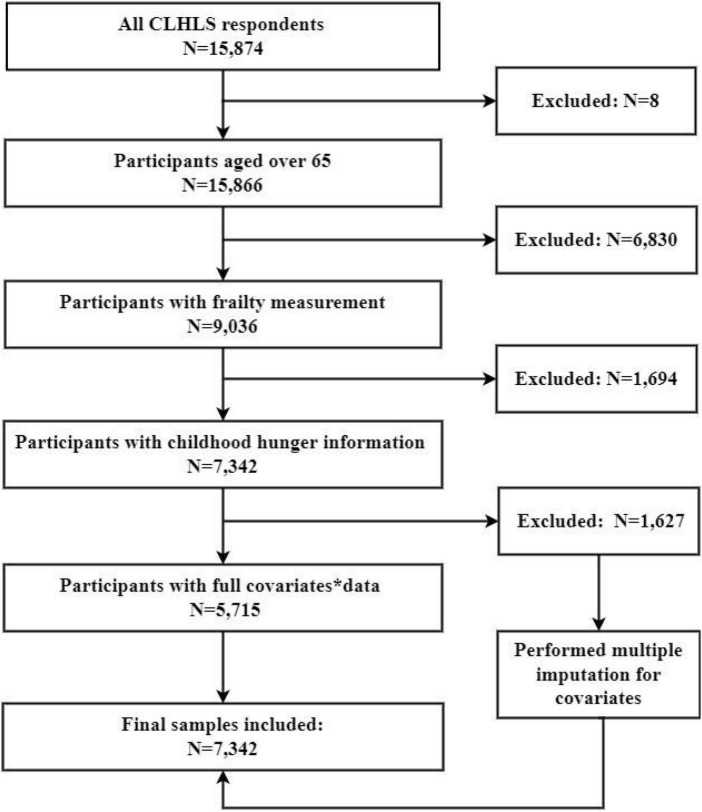
Flowchart on the sample selection and exclusion. *Covariates: residence, marriage, living arrangements, education, occupation, smoking, drinking, exercise, social and leisure activity index, dietary pattern, and nutrition status, financial support, insurance status.

A total of 15,874 participants were interviewed in the 2018 CLHLS survey. Among them, the proportion of the senior population (≥ 80 years old) was 65.7%. The inclusion criteria were as follows: (1) participant aged 65 years old or above; (2) complete information on frailty index and childhood hunger was collected. After excluding 8,524 participants due to missing data on key variables (6,830 with missing frailty data, and 1,694 with missing childhood hunger information), 7,342 participants aged 65 years or above finished the survey and had complete information on the frailty index and childhood hunger. Moreover, 5,700 had complete covariates data, and 1,642 had missing data on covariates (137 with missing residence, 64 with missing marriage, 127 with missing living arrangements, 118 with missing education, 131 with missing occupation, 609 with missing financial support, 175 with missing insurance status, 66 with missing smoking, 74 with missing drinking, 88 with missing exercise, 79 with missing social and leisure activity index, 152 with missing dietary patterns, and 246 with missing nutritional status). The samples having answers of “I don’t know/have no idea” in key variables were excluded in this study. Missing data were mainly due to no answer to key variables. To control bias from missing data, we managed missing data using multiple imputations, which is a relatively flexible and general purpose approach to dealing with missing data (10). Finally, a total of 7,342 participants were analyzed in this study (Figure 1). The missing participants were more likely to be female, aged 80 years or above, illiterate, have other marital statuses, living with household members, financially dependent on others, non-smokers, non-drinkers, performing no exercise, and have a low Body Mass Index (BMI).

### Frailty index

The frailty index (FI) is a mathematical model based on the accumulation of deficits, which can include any symptom, sign, disease, laboratory abnormality, or disability (11). Following the standard procedure proposed by Searle et al., we constructed the FI using 44 health deficits, including daily life events, chronic illness, and psychological functioning (Table 1). Although different numbers of deficits were used to construct each FI, the pattern of frailty with age remained consistent as long as the major domains of health, such as activities of daily living, were included (12). The deficits in the present study were comparable with those of other studies (12), with a Cronbach’s alpha of 0.868. Each deficit variable was dichotomized or multicut and mapped to the 0–1 interval (e.g., routine task-bathing, with “no assistance” coded as 0, “partial assistance” coded as 0.5, and “need assistance” coded as 1) to indicate its severity. The sum of all deficits (*n* = 44) was then used to calculate the FI, which ranged from 0 to 1. We divided the FI score into three levels of variables: non-frail (FI ≤ 0.10), pre-frail (0.10 < FI ≤ 0.21), and frail (FI > 0.21) (13).

**TABLE 1:** Variables for constructing frailty index in the 2018 waves of Chinese Longitudinal Healthy Longevity Survey.

Number	Variables	Values
1	Feel useless with age	Never = 0; seldom = 0.25; sometimes = 0.5; often = 0.75; always = 1
2	Feel lonely and isolated	Never = 0; seldom = 0.25; sometimes = 0.5; often = 0.75; always = 1
3	Feel fearful or anxious	Never = 0; seldom = 0.25; sometimes = 0.5; often = 0.75; always = 1
4	Keep my belongings neat and clean	Always = 0; often = 0.25; sometimes = 0.5; seldom = 0.75; never = 1
5	Self-reported health	Very good = 0; good = 0.25; so so = 0.5; bad = 0.75; very bad = 1
6	Do you feel any change in your health since	Much better = 0; slightly better = 0.25; almost the same = 0.5;
	The last year?	Slightly worse = 0.75; much worse = 1
7	Make own decision	Always = 0; often = 0.25; sometimes = 0.5; seldom = 0.75; never = 1
8	Bathing	Without assistance = 0; partial assistance = 0.5; need assistance = 1
9	Dressing	Without assistance = 0; partial assistance = 0.5; need assistance = 1
10	Toileting	Without assistance = 0; partial assistance = 0.5; need assistance = 1
11	Transferring	Without assistance = 0; partial assistance = 0.5; need assistance = 1
12	Continence	Without assistance = 0; partial assistance = 0.5; need assistance = 1
13	Feeding	Without assistance = 0; partial assistance = 0.5; need assistance = 1
14	Able to go outside to visit neighbors?	Yes = 0; a little difficult = 0.5; not able to do so = 1
15	Able to go shopping by yourself?	Yes = 0; a little difficult = 0.5; not able to do so = 1
16	Able to make food by yourself?	Yes = 0; a little difficult = 0.5; not able to do so = 1
17	Able to wash clothes by yourself?	Yes = 0; a little difficult = 0.5; not able to do so = 1
18	Able to walk 1 km?	Yes = 0; a little difficult = 0.5; not able to do so = 1
19	Able to carry 5-kg weight?	Yes = 0; a little difficult = 0.5; not able to do so = 1
20	Able to crouch and stand for three times?	Yes = 0; a little difficult = 0.5; not able to do so = 1
21	Able to take public transport?	Yes = 0; a little difficult = 0.5; not able to do so = 1
22	Visual function	Can see and distinguish the break in the circle = 0; can see but not
		Distinguish the break in the circle = 0.33; cannot see = 0.67; blind = 1
23	Hand behind neck	Both = 0; right = 0.5; left = 0.5; neither = 1
24	Hand behind lower back	Both = 0; right = 0.5; left = 0.5; neither = 1
25	Raise arms upright	Both = 0; right = 0.5; left = 0.5; neither = 1
26	Able to stand up from sitting in a chair	Yes, without using hands = 0; yes, using hands = 0.5; no = 1
27	Able to pick up a book from the floor	Yes, without using hands = 0; yes, using hands = 0.5; no = 1
28	Of times suffering from serious illness	Not applicable = 0; one serious illness = l; two or more serious
	In the past 2 years	Illnesses = 2
29	Suffering from hypertension?	No = 0; yes = 1
30	Suffering from diabetes?	No = 0; yes = 1
31	Suffering from heart disease?	No = 0; yes = 1
32	Suffering from stroke or cardiovascular disease?	No = 0; yes = 1
33	Suffering from bronchitis, emphysema,	No = 0; yes = 1
	Pneumonia, and asthma?	
34	Suffering from tuberculosis?	No = 0; yes = 1
35	Suffering from cataract?	No = 0; yes = 1
36	Suffering from cancer?	No = 0; yes = 1
37	Suffering from glaucoma?	No = 0; yes = 1
38	Suffering from gastric or duodenal ulcer?	No = 0; yes = 1
39	Suffering from Parkinson’s disease?	No = 0; yes = 1
40	Suffering from bedsore?	No = 0; yes = 1
41	Suffering from arthritis?	No = 0; yes = 1
42	Suffering from dementia?	No = 0; yes = 1
43	Was interviewee able to hear?	Yes, without hearing aid = 0; yes, but needs hearing aid = 0.33;
		Partly, despite using hearing aid = 0.67; no = 1
44	The health of interviewee rated	Surprisingly healthy = 0; relatively healthy = 0.33; moderately
	By interviewer	ill = 0.67; very ill = 1

### Childhood hunger

Childhood hunger was measured by the question “Did you often go to bed hungry as a child?” The responses included “yes” or “no.”

### Explanatory variables

Sociodemographic characteristics included age (age 65–79 vs. age 80 +), sex (male vs. female), and residence (rural vs. urban). Family/social support included marriage and living arrangements. Marriage was divided into married and other (including separated, divorced, widowed, and unmarried). Living arrangements were classified as follows: living alone, living with household members, and living in an institution. Socioeconomic status included education, occupation, and financial support. Education was divided into illiterate, primary school, junior high school, and high school and above. Occupations before 60 years old were allocated into two categories: agricultural work, which was coded as 0, and non-agricultural work, which was coded as 1. Financial support included financial dependence (coded as 0) and financial independence (coded as 1). Participants’ financial independence included work and retirement wages, and financial dependence included participants’ financial dependence on other family members. Insurance status was categorized into the New Rural Cooperative Medical Scheme (NRCMS), Urban Basic Medical Insurance Scheme (UBMIS) (including urban resident basic medical insurance and urban employee basic medical insurance), others (including commercial medical insurance and public free medical services), and no insurance.

Health behaviors included smoking (yes or no), drinking per day (liang) (including 0, 0 < –1, 1 < –2, and > 2), exercise (yes or no), social and leisure activity index, dietary patterns, and nutritional status. The social and leisure activity scores were calculated for eight activity types (whether a respondent gardened, practiced Tai Chi, participated in square dancing, kept poultry or pets, read, played Mahjong or cards, listened to the radio or watched TV, and participated in community social activities). We scored each activity 1 for “never,” 2 for “sometimes,” and 3 for “almost every day.” Scores ranged from 8 to 24, with 14 or less being defined as a low social and leisure activity level, and higher scores indicating more leisure activities. Dietary patterns were classified as unfavorable, intermediate, or favorable through a simplified healthy eating index based on the frequency of intake of five food groups: fish, vegetables, fruits, tea, and bean products, which have been shown to be associated with frailty. The intake scores for these five food groups were summed and divided into three categories: unfavorable: 0–4; intermediate: 5–6; favorable: 7–10. BMI reflects nutritional status. According to the Global Leadership Initiative on Malnutrition (GLIM) criteria (14), the BMI cut-off for malnutrition risk is < 18.5 kg/m^2^ if the participant is aged < 70 years, and < 20 kg/m^2^ if the participant is aged ≥ 70 years; or else, the participant is identified as normal BMI.

### Statistical analysis

Multiple imputation was used to adjust for selection bias and information loss. In the multiple imputation strategy, 50 iterations were used to impute missing data, and five imputed datasets were generated using predictive mean matching. The results were pooled over all five sets using Rubin’s rules. We present the descriptive statistics, and the results are expressed as the number of categorical variables (proportions). The relationship between childhood hunger and sociodemographic characteristics, family/social support, socioeconomic status, insurance status, and health behaviors was analyzed using chi-square tests, and the same process was applied to frailty. Covariates with three or more classifications were analyzed for their differences using a chi-square test of partitioning. Bonferroni correction was used for multiple comparisons. *P* < 0.017 was considered statistically significant for a two-way comparison between the 3 groups and *P* < 0.008 was considered statistically significant for a two-way comparison between the 4 groups. Mean and standard deviation (SD) were used to describe age. We evaluated multicollinearity among covariates, and the largest variance inflation factor was < 2, suggesting no multicollinearity biases in the models. The relationship between childhood hunger and frailty was evaluated by ordinal logistic regression analysis. Subgroup analyses were then conducted by stratifying variables. Finally, we explored whether age, financial support, and insurance status were potential moderators of this relationship, and we added an interaction term to test for a moderating effect. All statistical analyses were conducted with SPSS 26.0. A *p*-value of < 0.05 was considered statistically significant.

## Results

As shown in Table 2, the sample was composed of 7,342 participants, comprising 3,420 males (46.6%) and 3,922 females (53.4%). The mean age of the study group was 82.99 (*SD* = 11.4) years. Of these participants, 42.1% were aged 65–79 years, 57.9% were aged ≥ 80 years, 46.0% were married, 82.9% resided in rural areas, 17.3% had high school and above, 61.2% did agricultural work, and 58.5% were financially dependent on others. A total of 1,888 (25.7%) were covered by UBMIS, 4,429 (60.3%) were covered by NRCMS, 229 (3.1%) were others, and 796 (10.9%) were not covered.

**TABLE 2:** Association of baseline characteristics with childhood hunger and frailty; data are expressed as number (prevalence) [n (%)].

Characteristics	Total n (%)	Childhood hunger n (%)	χ^2^	Frailty n (%)	χ^2^
*Total*	7,342	5,170 (70.42)		2,150 (29.28)	
*Age group* (years)			13.211***		1437.747***
65–79	3,089 (42.07)	2,105 (68.15)		253 (8.19)	
80 +	4,253 (57.93)	3,065 (72.07)		1,897 (44.60)	
*Sex*			6.374*		192.327***
Female	3,922 (53.42)	2,811 (71.67)		1,377 (35.11)	
Male	3,420 (46.58)	2,359 (68.98)		773 (22.60)	
*Residence*			517.174***		17.609***
Rural	6,085 (82.88)	4,620 (75.92)		1,720 (28.27)	
Urban	1,257 (17.12)	550 (43.76)		430 (34.18)	
*Marital status*			14.425***		761.973***
Married	3,375 (45.97)	2,302 (68.22)		508 (15.04)	
Others	3,967 (54.03)	2,868 (72.28)		1,642 (41.40)	
*Living arrangement*			16.685***		182.672***
With household member(s) (1)	6,123 (83.39)	4,348 (71.02)		1,884 (30.76)	
In an institution (2)	197 (2.69)	114 (57.85)^a^		106 (53.90)^a^	
Alone (3)	1,022 (13.92)	708 (69.25)^b^		160 (15.66)^ab^	
*Education*			607.065***		514.832***
High school and above (1)	1,273 (17.34)	568 (44.64)		267 (20.97)	
Junior high school (2)	1,130 (15.39)	752 (66.54)^a^		193 (17.09)	
Primary school (3)	1,766 (24.05)	1,259 (71.30)^ab^		400 (22.68)^b^	
Illiterate (4)	3,173 (43.22)	2,591 (81.65)^abc^		1,290 (40.64)^abc^	
*Occupation*			463.701***		13.535**
Agricultural work	4,492 (61.19)	3,574 (79.55)		1,253 (27.89)	
Non-agricultural work	2,850 (38.81)	1,596 (56.01)		897 (31.49)	
*Financial support*			390.197***		201.103***
Financial dependence	4,296 (58.52)	3,406 (79.28)		1,462 (34.02)	
Financial independence	3,046 (41.48)	1,764 (57.92)		688 (22.60)	
*Insurance status*			499.079***		49.934***
NRCMS (1)	4,429 (60.32)	3,539 (79.90)		1,189 (26.84)	
UBMIS (2)	1,888 (25.71)	1,015 (53.77)^a^		581 (30.76)^a^	
Others (3)	229 (3.12)	125 (54.71)^a^		80 (35.08)^a^	
No (4)	796 (10.85)	491 (61.63)^ab^		300 (37.72)^ab^	
*Smoking*			5.186*		148.532***
Yes	1,212 (16.51)	887 (73.14)		201 (16.58)	
No	6,130 (83.49)	4,283 (69.88)		1,949 (31.80)	
*Drinking per day (liang)*			1.358		125.001***
0 (1)	5,309 (72.30)	3,718 (70.04)		1,710 (32.21)	
0 < –1 (2)	641 (8.73)	457 (71.34)		172 (26.82)^a^	
1 < –2 (3)	618 (8.43)	443 (71.68)		131 (21.21)^a^	
>2 (4)	774 (10.54)	552 (71.25)		137 (17.72)^ab^	
*Exercise*			41.657***		452.748***
Yes	2,623 (35.72)	1,726 (65.80)		402 (15.32)	
No	4,719 (64.28)	3,444 (72.98)		1,748 (37.04)	
*Social and leisure activity level*			58.064***		387.330***
High	1,248 (17.00)	767 (61.45)		119 (9.55)	
Low	6,094 (83.00)	4,403 (72.25)		2,031 (33.33)	
*Dietary pattern*			197.711***		159.645***
Unfavorable (1)	2,828 (38.52)	2,207 (78.04)		998 (35.31)	
Intermediate (2)	3,161 (43.05)	2,192 (69.35)^a^		841 (26.61)^a^	
Favorable (3)	1,353 (18.43)	771 (56.99)^ab^		311 (22.95)^ab^	
*BMI*			33.582***		146.387***
Low BMI	1,855 (25.27)	1,405 (75.72)		730 (39.36)	
Normal BMI	5,487 (74.73)	3,765 (68.62)		1,420 (25.88)	

UBMIS represents urban basic medical insurance scheme, NRCMS represents new rural cooperative medical scheme, Others represents commercial medical insurance and public free medical services, BMI represents body mass index.

*P < 0.05, **P < 0.01, ***P < 0.001.

^a^ represents significant difference in childhood hunger or frailty compared with the group (1); ^b^ represents significant difference in childhood hunger or frailty compared with the group (2); ^c^ represents significant difference in childhood hunger or frailty compared with the group (3).

Overall, the prevalence of childhood hunger was 70.4%, with 68.2% in the 65–79 years group and 72.1% in the ≥ 80 years group (Table 2). Hunger in childhood was more likely to be experienced by individuals with the following characteristics: female, residents of rural areas, other marital statuses, living with household members, illiterate, had been an agricultural worker, financial dependence on others, having NRCMS, smoking, performing no exercise, low social and leisure activity levels, unfavorable dietary patterns, and low BMI. There were no significant differences between childhood hunger and drinking.

Table 2 also shows the prevalence of frailty according to participants’ characteristics. Of the 7,342 eligible participants, 2,465 (33.6%) were non-frail, 2,727 (37.1%) were pre-frail and 2,150 (29.3%) were frail. Participants with frailty symptoms were likely to have the following characteristics: older, female, living in urban areas, other marital statuses, living in an institution, illiterate, non-agricultural work, financial dependence on others, without insurance, non-smokers, non-drinkers, performing no exercise, low social and leisure activity levels, unfavorable dietary patterns, and low BMI.

As shown in Table 3, older adults who experienced childhood hunger were more likely to have frailty than those who did not (OR = 1.15, 95% CI: 1.05–1.26) in the crude model. Further adjustment for sociodemographic characteristics, family/social support, socioeconomic status, insurance status, and health behaviors did not affect the relationship (OR = 1.13, 95% CI: 1.02–1.26). Considering differences in age, sex, residence, socioeconomic status, and insurance status in relation to frailty, *post hoc* analyses stratified by age, sex, residence, socioeconomic status, and insurance status were conducted. In the final model, the association of childhood hunger with frailty was found in the 65–79 years group (OR = 1.21, 95% CI: 1.03–1.43), women (OR = 1.25, 95% CI: 1.08–1.45), rural residents (OR = 1.16, 95% CI: 1.03–1.31), agricultural work (OR = 1.16, 95% CI: 1.00–1.34), those with financial dependence (OR = 1.18, 95% CI: 1.02–1.37), and NRCMS (OR = 1.35, 95% CI: 1.16–1.56). In the crude model, childhood hunger was significantly associated with lower odds of frailty in high school and above and financial independence. However, in the final model, the difference was small and not statistically significant.

**TABLE 3:** The association of childhood hunger and frailty stratified by age, sex, residence, socioeconomic status, insurance status.

Characteristics	Crude model OR (95% CI)	Final model OR (95% CI)
*Childhood hunger*	1.15 (1.05, 1.26)**	1.13 (1.02, 1.26)*
Stratified by age *group*		
65–79	1.15 (0.99, 1.33)	1.21 (1.03, 1.43)*
80 +	1.04 (0.91, 1.18)	1.05 (0.92, 1.21)
Stratified by *sex*		
Female	1.27 (1.12, 1.45)***	1.25 (1.08, 1.45)**
Male	0.98 (0.86, 1.12)	1.03 (0.88, 1.20)
Stratified by *residence*		
Urban	1.03 (0.84, 1.26)	1.11 (0.87, 1.41)
Rural	1.28 (1.14, 1.43)***	1.16 (1.03, 1.31)*
Stratified by *education*		
High school and above	0.64 (0.52, 0.79)***	1.01 (0.79, 1.29)
Junior high school	1.02 (0.81, 1.28)	1.22 (0.94, 1.59)
Primary school	0.82 (0.68, 1.00)	1.15 (0.93, 1.42)
Illiterate	1.14 (0.96, 1.35)	1.14 (0.95, 1.37)
Stratified by *occupation*		
Agricultural work	1.37 (1.20, 1.57)***	1.16 (1.00, 1.34)*
Non-agricultural work	1.03 (0.89, 1.18)	1.17 (0.99, 1.37)
Stratified by *financial support*		
Financial dependence	1.28 (1.11, 1.47)**	1.18 (1.02, 1.37)*
Financial independence	0.77 (0.67, 0.88)***	1.11 (0.94, 1.30)
Stratified by *insurance status*		
NRCMS	1.52 (1.33, 1.75)***	1.35 (1.16, 1.56)***
UBMIS	0.97 (0.82, 1.15)	0.99 (0.82, 1.20)
Others	0.89 (0.55, 1.44)	1.37 (0.73, 2.55)
No	1.11 (0.84, 1.45)	0.93 (0.68, 1.28)

OR represents the odds ratio, 95% CI represents 95% confidence intervals, UBMIS represents urban basic medical insurance scheme, NRCMS represents new rural cooperative medical scheme, Others represents commercial medical insurance and public free medical services.

*P < 0.05, **P < 0.01, ***P < 0.001.

The final model is adjusted for age, sex, residence, marital status, living arrangement, education, occupation, financial support, insurance status, smoking, drinking per day, exercise, social and leisure activity index, dietary pattern, and BMI.

Given that age, financial support, and insurance status could mediate the relationship of childhood hunger with frailty, we tested the interaction between childhood hunger and age, financial support, and insurance status (Table 4). The results showed that the effects of childhood hunger on frailty were partially mediated by age, financial support, and insurance status. The 80 years or older group (OR = 3.61, 95% CI: 3.22–4.04) were significantly associated with higher odds of frailty. Participants with hunger in childhood who were 80 years or older (OR = 0.80, 95% CI: 0.65–0.98) had a lower odds ratio of frailty. NRCMS (OR = 0.66, 95% CI: 0.56–0.77) was significantly associated with lower odds of frailty. However, NRCMS (OR = 1.42, 95% CI: 1.02–1.98) showed an increased odds ratio of childhood hunger-related frailty.

**TABLE 4:** Effect of the interaction between childhood hunger and age, and insurance status on frailty.

Characteristics	Crude model OR (95% CI)	Final model OR (95% CI)
*Childhood hunger*	1.15 (1.05, 1.26)**	1.13 (1.02, 1.26)*
*Age group* (Ref. = *65–79 years*)		
80 +	5.95 (5.40, 6.55)***	3.61 (3.22, 4.04)***
Childhood hunger × Age group		0.80 (0.65, 0.98)*
*Insurance status* (Ref. = *No*)		
NRCMS	0.67 (0.58, 0.77)***	0.66 (0.56, 0.77)***
UBMIS	0.78 (0.67, 0.91)**	1.07 (0.90, 1.27)
Others	0.79 (0.60, 1.04)	1.05 (0.78, 1.42)
Childhood hunger × Insurance status		
NRCMS		1.42 (1.02, 1.98)*
UBMIS		0.91 (0.65, 1.28)
Others		1.06 (0.59, 1.91)

OR represents the odds ratio, 95% CI represents 95% confidence intervals, Ref. represents reference, UBMIS represents urban basic medical insurance scheme, NRCMS represents new rural cooperative medical scheme, Others represents commercial medical insurance and public free medical services.

*P < 0.05, **P < 0.01, ***P < 0.001.

Multiple logistic regression analysis was applied to estimate the OR and 95% CI for frailty. The final model is adjusted for age, sex, residence, marital status, living arrangement, education, occupation, financial support, insurance status, smoking, drinking per day, exercise, social and leisure activity index, dietary pattern, and BMI.

## Discussion

This study used a large, nationally representative sample of older Chinese individuals to evaluate the association between childhood hunger and frailty in old age. The findings showed that older adults who often experienced hunger as children had a significantly higher risk of frailty, especially those with low socioeconomic status, suggesting that more light should be shed on policies or interventions to end children and adolescents’ hunger in consideration of the socioeconomic status, providing a better understanding of the determinants of healthy longevity.

Previous studies (5) explored the relationship between food deprivation in early life and risk of frailty in older Chinese adults aged 45 years and above using the data from the China Health and Retirement Longitudinal study, and showed that exposure to food deprivation in childhood was also significantly associated with frailty. Experiencing prolonged hunger and poor health and growing up in a family with poor socioeconomic conditions can have a strong and lasting impact on later health (15). Our findings suggested that older adults who experienced childhood hunger were more likely to experience frailty than those who did not. People who experience malnutrition in their early years are at a higher risk of subsequently developing metabolic syndrome in a nutrient-rich environment due to metabolic maladjustment (6). Exposure to hunger early in life was found to increase the probability of being overweight and depressed in old age (4). Our findings suggested that nutrition is closely related to frailty syndrome, and all frailty criteria are more or less influenced by poor dietary habits. Studies have shown an association between frailty and specific components of the diet, including protein and energy intake, as well as the intake of specific micronutrients (16). This evidence indicates that nutritional status in early life is closely linked to health conditions in old age. Thus, improving nutritional status early in life should be prioritized to control the increasing trend of chronic non-communicable diseases (17).

Our findings suggested that older adults who were hungry in childhood, females, rural residents, agricultural workers, those with NRCMS, and those who were financially dependent had a higher odds ratio of frailty, which is consistent with earlier studies (18). Generally, the prevalence of frailty increased with the increase of age. The aging process and longevity have a direct impact on frailty status; thus, frailty is more prevalent in older people (19). However, our findings also showed that people who were 80 years or older and experienced hunger in childhood had a lower odds ratio of frailty. A potential reason for this finding relates to survivor bias, as individuals who were 80 years or older may already have died if they had a poor health status and from a low socioeconomic status (20).

Our findings indicated that women who experienced childhood hunger had an aggravated possibility of frailty. A previous study reported that childhood hunger has a stronger effect on physical health outcomes in women than in men (21). The discrepancy may be associated with the noticeable gender difference to pro-male bias in the Chinese culture (4). The apparent increase in the prevalence of frailty with age among women may be in part a result of frail women outliving frail men (22). This survival advantage in women is often linked to a higher prevalence of disability and poor health status (22). Additionally, women are also more prone to developing psychosocial disorders associated with frailty due to their lifetime stressors, poverty, and loneliness at the end of life (23).

Our findings showed that rural residence was significantly associated with childhood hunger. Rural residents had a significantly higher risk of malnutrition than urban residents (18), and differences in frailty and life expectancy were found between rural and urban older adults. Urban residents may have an advantageous educational system compared to rural residents, which may influence individual health outcomes (24). Rural residence with low levels of education might increase the risk of frailty in older adults. This finding may reflect the impacts of regional differences in socioeconomic and environmental attributes or access to health care between two populations (25). Our analysis found that having UBMIS as the main payment method was effective in alleviating healthcare costs for older Chinese individuals compared to out-of-pocket spending. Our findings showed that participants who experienced hunger in childhood and with UBMIS had a lower odds ratio of frailty. However, NRCMS showed an increased odds ratio of childhood hunger-related frailty. Previous studies have reported that the actual reimbursement rate for UBMIS enrollees was higher than that for NRCMS enrollees in China (26). The NRCMS has the weakest financial security, which is consistent with other scholars’ studies. The NRCMS covers a greater proportion of the rural population, who are also the most vulnerable group for non-communicable diseases (27). In China, the rural population has more restricted access to health services and a heavier financial burden than urban residents (28). Higher income individuals are reimbursed more frequently than lower income individuals, who are less healthy, and inequalities in welfare exacerbated health inequities (29). There is a gap between nominal and actual reimbursement rates, and the NRCMS has not significantly reduced this gap (30).

Previous studies interviewed 13,185 individuals aged 65–99 years and found that childhood experiences of hunger affect socioeconomic status in adult life, which, in turn, can affect health outcomes in older adults (31). Our findings showed that high educational level and financial independence may reduce the probability of childhood hunger-mediated frailty in older age. Education builds an individual’s knowledge and skills, determines future attitudes and behaviors, and helps people achieve a better occupational class and higher economic status (32). Although education and income do not directly affect the pathophysiology of frailty, they may interfere with the lifestyle of the individual and influence the development of frailty (33). Thus, education is also a good social predictor of frailty, and reflects childhood circumstances and attained adult socioeconomic status (34). Our findings also identified that financial dependence was significantly associated with both childhood hunger and frailty, and older adults with childhood hunger who were financially dependent on others had a higher odds ratio of frailty. Poor financial security is one of the most important risk factors of frailty in old age (35). Older people with a low income might choose to live alone, which can lead to an increased risk of developing frailty because they may be less likely to have the ability to meet their daily needs (36).

As older people become frailer, their level of physical activity decreases, and this lowered physical activity in turn provokes a vicious cycle, which can make the frail older people become frailer (37). Our findings suggested that participants with frailty symptoms were likely to have no exercise and low social and leisure activity levels. Social participation in older people directly increases social interactions, which has the potential to result in decreased cognitive decline and decreased risk of having depression; moreover, it also increases physical activities, which decreases the risk of developing frailty (38).

Our study showed that high prevalence of frailty was associated with low BMI. Being underweight or obese can increase the risk of frailty and sarcopenia (39). Healthy nutrition may alleviate the risk of being obese or underweight, further decreasing the risk of frailty (40). Older people with normal BMI had a relatively low prevalence of frailty in our study. In fact, in addition to the population, the setting also seems to determine the relationship between BMI and adverse outcomes in older adults (41). A lower BMI would be more favorable in community dwelling older adults in terms of frailty or functionality (42), but the opposite was reported to be true for nursing home residents (43). Maintaining a healthy BMI in older adults is important for maintaining healthy nutritional status and skeletal muscle mass (44). Notably, non-smokers and non-drinkers were more likely to be frail in our study. It is possibly explained by abstainer/quitter bias; for example, people might have been advised not to smoke or drink because of poor health (45).

## Strengths and limitations

This study investigated the relationship of childhood conditions with the aging process and health status in older adults in the context of socioeconomic status based on a large representative sample of centenarians in China, providing a better understanding of the determinants of healthy longevity. Our research has several limitations. First, frailty index represents the cumulative deficit model and has been criticized for being a disease checklist rather than an assessment tool for physiological reserves. This study adopted a more detailed definition of frailty index using 44 health deficits as top studies constructing a frailty index (13); actually, there are appropriate and useful tools for identifying “true frailty,” e.g., Fried frailty scale (46) and SARC-F questionnaire (47). Second, this study determined the nutritional status of older people according to the GLIM criteria. However, it does not take gender difference into account, since it is well known that female gender is associated with higher fat mass than male. Third, differences in demographic characteristics, social support, socioeconomic status, and health behaviors between the missing participants and study participants may have influenced our results. Finally, frailty status might change over time, and we could not explore the impact of relevant risk factors on the frailty trajectory. More longitudinal studies are needed to identify the determinants of frailty progression or remission in older adults.

## Conclusion

Exposure to hunger during childhood is linked to frailty among older adults, and age, financial support, and insurance status may mediate this relationship. In early life, nutrition-targeted interventions and policies should be implemented to address hunger, and universal access to education should be promoted to reduce the socioeconomic status gap that accumulates in old age. In old age, socio-economically relevant strategies to control medical expenses for older people and to improve the reimbursement rate for NRCMS are beneficial in reducing inequality in frailty.

## Data availability statement

The datasets presented in this study can be found in online repositories. The names of the repository/repositories and accession number(s) can be found below: https://opendata.pku.edu.cn/dataverse/CHADS.

## Author contributions

TG and HL conceived the study. TG and SH analyzed the data. GM, QS, and MZ helped in interpreting the data. TG wrote the manuscript. All authors reviewed and approved the final version of the manuscript.

## Abbreviations

CLHLS: Chinese Longitudinal Healthy Longevity Survey
GLIM: Global Leadership Initiative on Malnutrition
FI: Frailty Index
OR: Odds Ratio
CI: Confidence Intervals
NRCMS: New Rural Cooperative Medical Scheme
UBMIS: Urban Basic Medical Insurance Scheme
BMI: body mass index.
